# 3-APTES on Dendritic Fibrous Mesoporous Silica Nanoparticles for the pH-Controlled Release of Corrosion Inhibitors

**DOI:** 10.3390/nano13182543

**Published:** 2023-09-11

**Authors:** Eleonora Marconi, Igor Luisetto, Gabriella Di Carlo, Maria Paola Staccioli, Simonetta Tuti, Luca Tortora

**Affiliations:** 1 LASR3 Surface Analysis Laboratory Roma Tre, Via della Vasca Navale 84, 00146 Rome, Italy; eleonora.marconi@roma3.infn.it; 2 National Institute for Nuclear Physics, INFN Roma, Tre Via della Vasca Navale 84, 00146 Rome, Italy; 3 Department of Sciences, Roma Tre University, Via della Vasca Navale 84, 00146 Rome, Italy; simonetta.tuti@uniroma3.it; 4 Department of Energy Technologies, ENEA C.R. Casaccia DTE-PCU-IPSE, S.P. 081 Via Anguillarese 301, S.M. di Galeria, 00123 Rome, Italy; igor.luisetto@enea.it; 5 Institute for the Study of Nanostructured Materials, National Research Council (ISMN-CNR), Via Salaria km 29.3, Monterotondo, 00015 Rome, Italy; gabriella.dicarlo@cnr.it (G.D.C.); mariapaola.staccioli@ismn.cnr.it (M.P.S.)

**Keywords:** mesoporous silica, nanocarrier, corrosion, 3-APTES, MBT, BTA

## Abstract

Mesoporous silica nanoparticles (MSNPs) are currently used in different fields like catalysis, nanomedicine, and conservation science, taking advantage of their high surface area. Here, we synthesized and functionalized mesoporous dendritic fibrous nanoparticles to realize a smart delivery system of protective agents for metals. Different MSNPs were obtained via the microemulsion method followed by a hydrothermal or refluxing treatment at different w/o ratios, times, and temperatures. Dendritic spherical silica nanoparticles with specific features such as an appropriate size (450 nm), a very large surface area (600 m^2^ g^−1^), and a high yield synthesis (86%) were selected for surface modification. The fiber surface of the selected MSNPs was functionalized with 3-aminopropyl triethoxysilane (3-APTES). 3-APTES works as a pH-driven “nanogate”, suppressing the immediate leakage of the total guest molecule load and modulating the release as a function of pH conditions. Surface-modified MSNPs were tested as a reservoir of the most diffused corrosion inhibitors: Mercaptobenzothiazole (MBT) and 1H-Benzotriazole (BTA); their release properties were studied in solutions with pH = 4 and 7. Functionalized and non-functionalized MSNPs showed a good loading efficiency of guest molecules (34–64%) and a pH-dependent release of the corrosion inhibitors on a timescale of several hours.

## 1. Introduction

Metals are, in general, present in different fields ranging from cultural heritage to building science as well as architecture. In some cases, like bridges or metal sculptures, the metals are exposed for centuries to extreme environmental conditions; consequently, considering their tendency to be oxidized, spontaneous corrosion phenomena occur with different mechanisms and kinetics [1,2]. The extent of corrosion can induce damages ranging from just a simple color variation to the complete alteration of the chemical, physical, and mechanical properties of the material [3,4]. Different factors can influence the corrosion process like the type of material, structural characteristics, presence of imperfections, conservation environment, and conservation history [5,6]. In recent years, the study of degradation phenomena in metal artifacts has received great attention [7], and new strategies were developed to arrest, or at least slow down, the deterioration processes related to corrosion phenomena [8]. Conventional methods usually involve the use of oxide layers [9] or organic coatings [10] as a protective passive barrier [11], which is becoming more and more frequently adopted as an alternative approach to cathodic/anodic protection [12]. One of the most widespread approaches to protect metal surfaces is based on the incorporation of corrosion inhibitors into a polymer matrix [13]. In the past, the corrosion inhibitors were directly added to the sol–gel matrix, leading to the production of passive barriers without any control over the inhibitor release and issues related to the fast deterioration of the sol–gel coating [14]. A new generation of anticorrosion coatings [15] adopts silica nanoparticles intending to control the corrosion inhibitor release [16]. Silica nanoparticles are widely employed in different fields thanks to the possibility of easily changing their shape and size [17]. In recent decades, several research works discussed the potential applications of silica nanoparticles in anticorrosive coatings, highlighting three main research subtopics: synthesis of tailored nanoparticles, development of corrosion inhibitor loading/gate/release systems, and applications in smart anticorrosive coatings. Developing nano-systems with different gating/release mechanisms is one of the most challenging fields due to the difficulty in realizing nanocarriers capable of responding to specific stimuli [11]. To achieve a self-healing coating, corrosion inhibitors are typically loaded in a nanocontainer using various synthetic strategies. Such engineered nanomaterials can then be dispersed, usually into organic coatings, resulting in a gradual or triggered release of the guest active molecules by external stimuli. This allows for an active repair of corroded sites on the metal [18].

Hollow silica nanoparticles were recently adopted as containers of corrosion inhibitors dispersed in a stable polymeric film [19]. Loading corrosion inhibitors into silica nanocontainers allows for the storage of functional molecules, keeping them separated and preventing prolonged interaction with the hosting material. Different encapsulation methods were reported in the literature, employing polymer containers, nanotubes, oxide nanoparticles, silica nanocontainers with polyelectrolyte shells, and layered double hydroxides [20,21,22,23,24]⁠.

Conversely, a different approach takes advantage of the high surface area of the mesoporous silica nanoparticles to store and release large amounts of functional molecules [25,26]. MSNPs are characterized by a high loading capacity, stability, tunable pore diameter, high surface area, and easy surface functionalization thanks to the presence of silanol groups [27,28,29,30].

A simple protocol based on the microemulsion method was conceived by Polshettiwar and coworkers to obtain dendritic mesoporous nanosilica (KCC-1) for application in catalysis [31]. Alternative synthetic approaches were developed using Teflon-sealed hydrothermal reactors [32,33,34] ⁠or refluxing systems [35]. Further synthesis protocols are present in the literature [36,37,38], where the formation mechanism of KCC-1 was modified to modulate the shape, size, and fiber density of the nanoparticles. The possibility to obtain mesoporous silica nanoparticles with high surface area was mainly exploited for drug delivery in the biomedical field [39,40]. In particular, several efforts were made to optimize their loading efficiency and release properties. The most consolidated approach is based on the use of amino silane groups as a gate-like system for a controlled release of the guest molecules [41]. The gatekeepers can be represented as linear molecules located on the mesoporous material surface, and their activity can be modulated using different stimuli such as pH, redox reactions, light exposure, or temperature variation. Polyamine molecules anchored on the pore outlets of the MSNPs were used to close the nanochannels exploiting the coulombic repulsions of the amino groups [42]. The combination of short polyamines and MSNPs led to a pH-driven and anion-controlled nano-supramolecular gate-like ensemble. The pH-controlled “open/close” mechanism most likely arises from hydrogen bonding interactions between amines (open gate) and coulombic repulsion between ammonium groups (closed gate). Recently, a similar approach was adopted for the functionalization of graphene-oxide-ordered mesoporous silica nanocontainers aimed at loading 1-H benzotriazole (BTA) as a corrosion inhibitor [43].

BTA and Mercaptobenzothiazole (MBT) are the most diffused and efficient corrosion inhibitors for the protection of copper-based works of art [44,45]. The MBT reaction mechanism is based on the presence of sulfur atoms outside the heterocyclic ring and nitrogen atoms on the heterocyclic ring that provides stable chemisorption on the metal surface, stopping the corrosion process on copper metal and carbon steel surfaces [46,47]. On the contrary, BTA molecules form a two-dimensional barrier that can protect the metal surface in aqueous media and various aggressive environments [48]. However, MBT and BTA are considered toxic and hazardous compounds [49,50]. Therefore, great efforts are focused on limiting the drawbacks related to the extensive release in the environment of these compounds by means of their confinement into nanocontainers.

In this work, we propose engineered amorphous mesoporous silica nanoparticles able to control the release of corrosion inhibitors in particular environments where corrosion phenomena are already in progress. A high-yield synthesis protocol to obtain MSNPs with peculiar features of KCC-1 was also developed. Dendritic MSNPs with large radial-fibrous morphology were selected and then functionalized with 3-APTES for a controlled release. MBT and BTA were tested as guest molecules by loading them onto functionalized and unfunctionalized MSNPs. The corrosion inhibitor release from the selected nanocarriers was evaluated in acidic and neutral solutions. A pH of 4 was selected to simulate a typical aggressive environment for metal, whereas a pH of 7 was used for comparison.

## 2. Materials and Methods

### 2.1. Reagents and Solutions

Tetraethyl orthosilicate (TEOS), cyclohexane, 1-penthanol, Cetylpyridinium Bromide hydrate (CPB), urea, 1H-benzotriazole (BTA), 2-Mercaptobenzothiazole (MBT), 3-Aminopropyl triethoxysilane (3-APTES), toluene anhydrous, ethanol, and water were purchased from Merck S.r.l. (Merck Life Science srl, Milan, Italy).

### 2.2. Synthesis of MSNPs

**Scheme 1:**
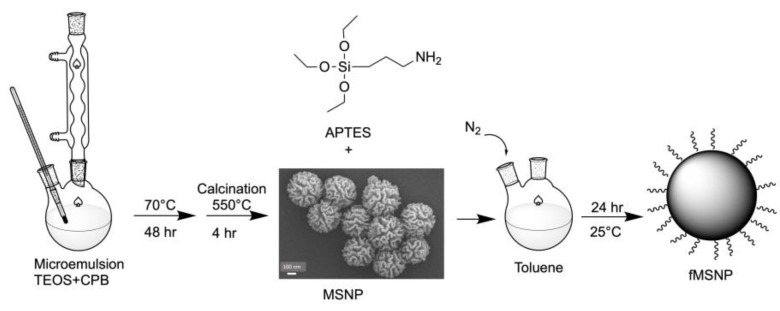
Synthesis and functionalization of MSNPs.

MSNPs were obtained via oil-in-water microemulsion with a volume ratio of 1:1. The oil phase was obtained by mixing 2.50 g of TEOS (12.00 mmol) and 1.50 mL of 1-pentanol (13.80 mmol) in 30.00 mL of cyclohexane (0.28 mol). The water phase was obtained by dissolving 1.00 g of CPB (2.60 mmol) and 0.60 g of urea (10.00 mmol) in 30.00 mL of water (1.66 mol). The final mixture was then heated using a hydrothermal reactor or a refluxing system. The hydrothermal synthesis was conducted at 120 °C at different holding times (2, 4, 16 h). The reflux synthesis was conducted at 70 °C for 24 or 48 h. The as-synthesized product was separated via centrifugation at 6000 rpm for 40 min and washed three times with abundant ethanol. Finally, the resulting white solid was dried and calcinated at 550° C for 4 h to remove the residual organic surfactant. In Scheme 1, the synthesis process is depicted.

### 2.3. Functionalization of MSNPs

**Scheme 2:**
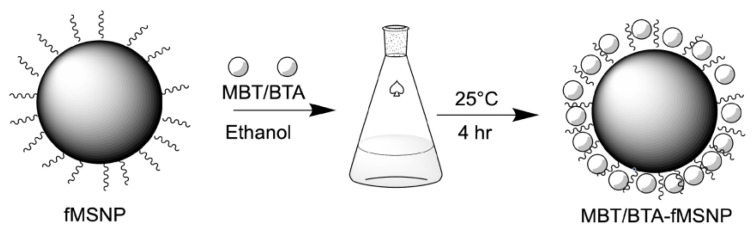
Loading strategy of the corrosion inhibitors in fMSNP.

MSNPs were functionalized with an aminic group using 3-APTES. First, 0.40 g of silica nanoparticles was dispersed in 10.00 mL of anhydrous toluene and stirred under nitrogen flow. A total of 1.00 mL of 3-APTES (4.00 mmol) was then injected into the dispersion. The mixture was stirred for 24 h at room temperature (see Scheme 2). MSNPs were separated via centrifugation at 6000 rpm for 20 min and washed in ethanol. The white powder was finally dried at 65 °C under a vacuum⁠. Hereinafter, the functionalized MSNPs will be referred to as fMSNPs.

### 2.4. Loading of MBT and BTA onto fMSNPs

The corrosion inhibitors BTA and MBT were loaded on both MSNP and fMSNP. Two different solutions were formulated. A total of 0.50 g of MBT (3.00 mmol) and BTA (4.20 mmol) were dissolved in ethanol (50.00 mL), showing a pale-yellow and transparent solution, respectively. The solution containing the corrosion inhibitors was then added with an equal volume (1:1 (*v*/*v*)) to a suspension solution of ethanol and silica nanoparticles (10.00 mg mL^−1^). The mixture was stirred for 4 h at room temperature. To gently remove the residual ethanol, the solid was filtered and dried at room temperature, obtaining a white powder for BTA-MSNP and MBT-MSNP.

On the contrary, the residual ethanol was removed from MBT-fMSNP and BTA-fMSNP via centrifuge at 6000 rpm for 10 min. The powder was dry overnight under the hood. In Table 1, all synthesized samples are summarized.

### 2.5. Characterization

Scanning Electron Microscopy images were collected using FE-SEM (Zeiss Sigma 300, Carl Zeiss Sigma NTS GmbH, Oberkochen, Germany) with an accelerating voltage of 10 kV. Elemental and mapping analysis was performed using the same instrument equipped with an EDS detector (Energy Dispersive X-ray Spectrometer—Bruker Nano GmbH, Berlin, Germany). ImageJ software was adopted to process SEM and TEM images in order to determine the average particle size of the MSNPs obtained through hydrothermal and refluxing methods.

The Zetasizer ULTRA instrument was employed for DLS measurements (Malvern PANalytical, Nottingham, UK). The setup consists of a He–Ne laser with a wavelength of 633 nm. Supernatant aliquots of each sample were analyzed at room temperature and then placed in a cylindrical glass cuvette with a 10 mm diameter. The DLS technique measures the particle self-diffusion coefficient (*D*). The self-diffusion coefficient is related to the hydrodynamic radius, DLS through the Stokes–Einstein equation:

$$D=\frac{k_{B}T}{6\pi \eta r}$$

The fluid viscosity (*η*) and the temperature (*T*) are input parameters that must be known a priori. The constant *k_B_* is the Boltzmann constant, and *r* is the radius of the spherical particle.

Attenuated Total Reflectance–Fourier transform infrared (FTIR-ATR) spectroscopy analysis (ThermoFisher^®^ Nicolet iS50, Waltham, MA, USA) was employed to evaluate the presence of corrosion inhibitors onto MSNPs. ATR-FTIR spectra were performed directly in 4000–400 cm^−1^ on powder samples milled. A beam of infrared light was passed through the ATR crystal diamond at an angle of 45°. The spectral resolution was 4 cm^−1^. A total of 100 scans were acquired for each spectrum.

Thermo Gravimetric Analysis (TGA/DSC1 Star System, Columbus, OH, USA) was used to quantify and detect the decomposition temperature of corrosion inhibitors deposited on the MSNPs. The sample mass was placed in an alumina crucible. Measurements were performed with an airflow of 70 mL min^−1^ using N_2_ as purge gas at 30 mL min^−1^. The weight change was recorded by holding the sample at 30 °C in an isothermal mode for 10 min and then increasing the temperature to 800 °C at a heating rate of 10 °C min^−1^.

N_2_ physisorption analysis was performed using ASAP 2020 (Micromeritics, Norcross, GA, USA). MSNPs were baked under a vacuum at 293 K before the analysis. The isotherms of the calcined MSNPs were obtained at 77 K. The Brunauer–Emmett–Teller (BET) method was employed to calculate the surface area, and the Barrett–Joyner–Halenda (BJH) method was used to calculate the pore size distribution.

The stimuli-responsive properties of MBT-fMSNP and BTA-fMSNP were investigated using UV-Vis spectroscopy (JASCO V 660 UV–Vis double-beam spectrophotometer, Jasco Corp., Tokyo, Japan). The release of MBT and BTA for MBT-MSNP and BTA-MSNP samples was also examined as a reference. A predetermined amount of fMSNP and MSNP, loaded with BTA and MBT, was placed in both distilled water and HCl solution at a pH of 4. A slow stirring was applied to the sample solution.

To study the release kinetics of the corrosion inhibitors, 3.0 mL of the supernatant mixture was collected at known time intervals for the analysis (Table S1). Then, the supernatant solution was measured using UV-Vis. The release of BTA and MBT was monitored through the changes in the intensity of characteristic absorption peaks at 260 nm and 320 nm, respectively. A calibration curve for BTA and MBT was obtained, reporting absorbance intensity values versus corrosion inhibitor concentrations in the range of 0.002 mg mL^−1^ and 0.01 mg mL^−1^ (Figure S1a,b).

## 3. Results and Discussion

### 3.1. MSNPs and fMSNPs

The synthesis of MSNPs was conceived to achieve particles with the smallest size, highest available surface area, and high yield. The silica nanoparticles were synthesized via microemulsion method followed by hydrothermal or refluxing treatment at different w/o ratios, times, and temperatures.

As listed in Table 2, the hydrothermal synthesis was conducted at w/o ratio of 1:1 and 1:2.3, heating the solution at 120 °C for 2, 4, and 16 h. On the other hand, the synthesis via the refluxing method was performed using a w/o ratio of 1:1 and heating, then microemulsion at 70 °C for 24 and 48 h. In the same Table, SEM and TEM images and the synthesis yield for the MSNPs are also reported. MSNPs obtained through hydrothermal synthesis (K-3, K-6, K-7, and K-9) show a compact core with thin fibers on the external part. Nanoparticles obtained through the refluxing method are instead characterized by spherical dendritic and concentrically stacked lamellar shapes (K-4 and K-5). For what regards the size, in all of the cases, the synthesis is characterized by a time-dependent structure evolution showing a gradual increase in size as a function of time. The particle size changes from 350 to 700 nm, holding the reaction time from 2 to 4 h (K6 and K3, respectively). By varying the solvent ratio from 1:1 to 1:2.3, the sphere size slightly decreased from 700 nm to 630 nm with an improvement of the synthesis yield from 60 to 80% (K7 and K9). The K-6 sample showed an average size of about ≈300 nm, but the synthesis yield was very low at about 4%. Nanoparticles obtained via the refluxing method are generally characterized by a smaller average size of about 400 nm and, in one case, a high-yield synthesis process.

It is important to note that a large part of MSNPs obtained through hydrothermal synthesis, here reported, could be considered unsuitable for application in protective coatings due to their large size. In fact, the use of protective films containing large nanoparticles could induce changes in the surface optical properties of the object modifying the aesthetic perception. We thus focused the attention on the synthesis and functionalization of MSNPs achieved through the refluxing method protocol (K-5 sample) for their appropriate size (450 nm) and very good yield (86%).

Scheme 1 represents the synthetic procedure for obtaining dendritic mesoporous silica (K-5 sample) and its functionalization with 3-APTES. The synthesis protocol is inspired by the well-known microwave-assisted hydrothermal technique where a water solution of CPB and urea is added to the organic solution of TEOS, and then, the mixture is irradiated with microwaves in a Teflon-sealed reactor [31]. Here, we have simplified the synthesis protocol by replacing the use of a microwave system at 400 W with a stirring of the microemulsion for 48 h at 70 °C in a refluxing system, as also reported elsewhere [35,36]. The resulting white solid is then separated, washed, and finally calcinated at 550 °C for 4 h.

**Figure 1:**
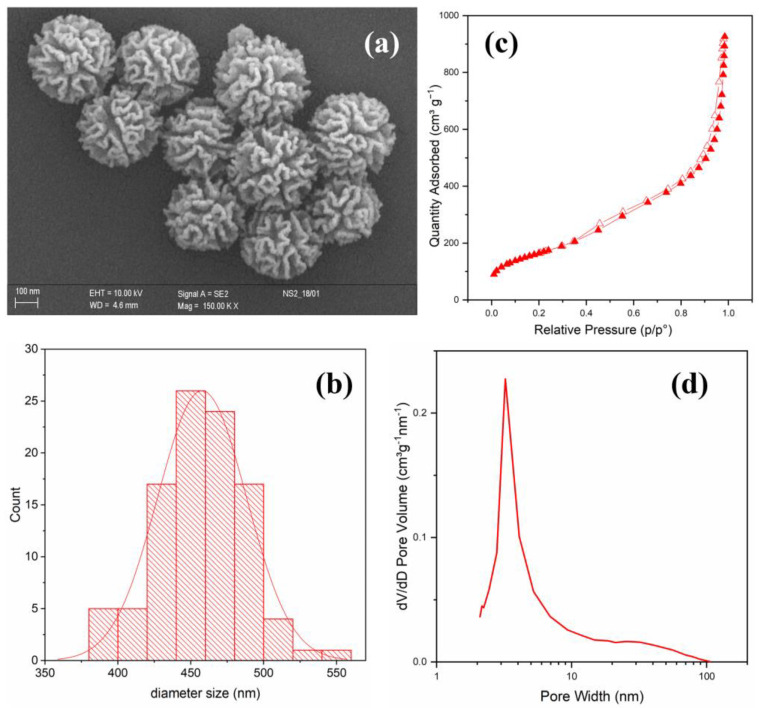
SEM image (**a**), size distribution histogram (**b**), and BET isotherms (**c**,**d**) of MSNPs (K-5 sample).

Synthetized MSNPs have a spherical shape with dendritic and fibrous morphology. SEM observations (Figure 1a) show fibers with a thickness of 15–20 nm and a distance between the fibers ranging from 30 nm up to 50 nm. MSNPs present a quite uniform size distribution with a diameter value in the range of 300–550 nm, as can be seen in the size distribution histogram, reported in Figure 1b. However, a large part of the MSNPs has a diameter size of around 450–470 nm. This was also confirmed via DLS analysis (Figure S2).

Surface area and pore-size distribution were investigated using the Brunauer–Emmett–Teller (BET) and Barrett–Joyner–Halenda (BJH) models, respectively. In Figure 1c, the N_2_ physisorption isotherms exhibit an IV-type curve, typical of mesoporous structures [37,51]. The BET surface area of calcined MSNPs is 592 m^2^ g^−1^, and the BJH total pore volume is 1.46 cm^3^ g^−1^. The pore size distribution curve reveals micropores with an average diameter of 8.74 nm (Table 2) peaked at 3.2 nm (Figure 1d). The structure of the synthesized MSNPs can still be considered comparable to KCC-1 in size and shape. On the contrary, fiber density, BET surface area, and BJH pore volume are slightly lower than KCC-1 [31]. The MSNP surface was then modified with an organoalkoxysilane by adding 3-APTES to a dispersion of silica nanoparticles in anhydrous toluene and stirring the solution under nitrogen flow for 24 h at room temperature (see Scheme 1). fMSNPs appear to be unaffected by the presence of the long-chain organosilicon compounds in terms of shape and fibrous morphology when observed with SEM. However, the functionalization was validated via DLS analysis reporting a shift of the hydrodynamic radius from 470 nm to 1121 nm (Figure S2). A strong decrease in surface area for the fMSNPs was also observed as a consequence of the functionalization, as shown in the data in Table 3, suggesting a substantial change in the surface composition.

### 3.2. MSNPs and fMSNPs + MBT and BTA

**Figure 2:**
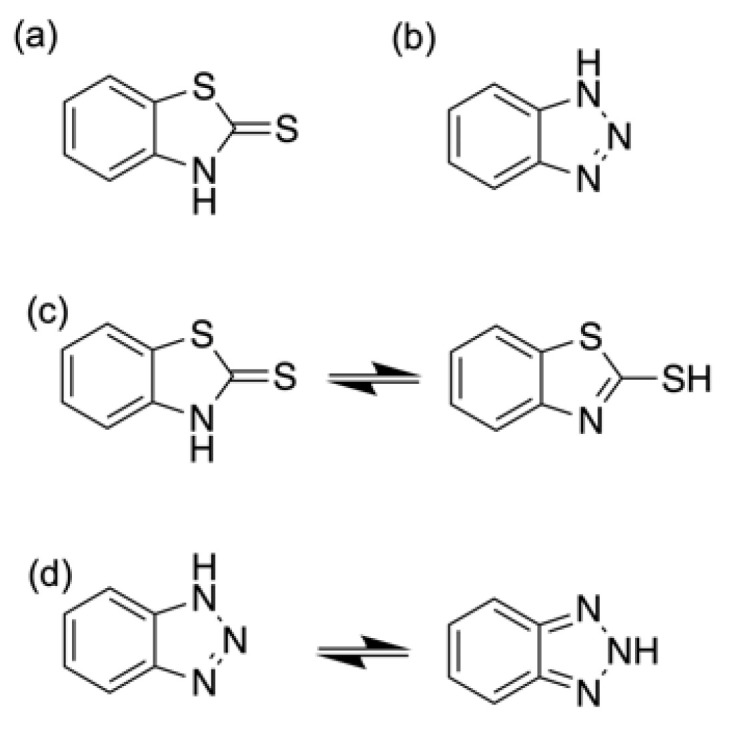
Structure of corrosion inhibitors (**a**) 2-Mercaptobenzothiazole (MBT) and (**b**) 1H-benzotriazole (BTA); tautomeric forms of MBT (**c**) and BTA (**d**).

MSNPs and fMSNPs were then loaded with the MBT (Figure 2a) and BTA (Figure 2b) corrosion inhibitors. Both MBT and BTA compounds can occur in two tautomeric forms reported in Figure 2c and Figure 2d, respectively. The loading process was achieved using simple physisorption for the MSNP samples and physisorption mediated with 3-APTES action for the fMSNP samples. The amide chain of 3-APTES physically entraps the corrosion inhibitor molecules into the pores of silica nanoparticles. The 3-APTES chains work like gatekeepers, managing the release of functional molecules as a function of pH. MBT and BTA were then put in contact with fMSNPs, leading to the formation of MBT-fMSNPs and BTA-fMSNPs. This was conducted by adding the solution containing the corrosion inhibitors to a solution of ethanol and silica nanoparticles (1:1 (*v*/*v*)) and stirring the mixture for 4 h at room temperature (Scheme 2).

**Figure 3:**
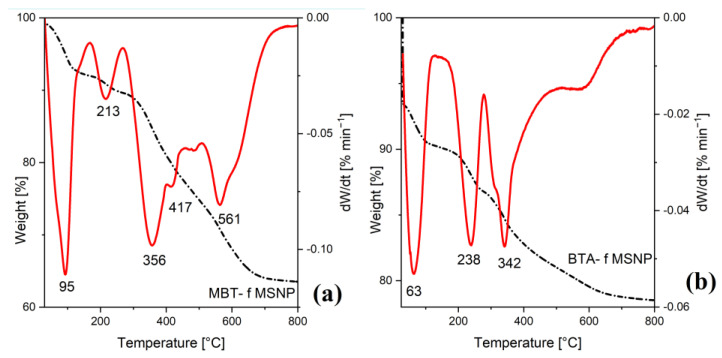
TGA (dash-dotted line) and DTG (solid line) of (**a**) MBT-fMSNP and (**b**) BTA-fMSNP.

In order to study the regulated release of the corrosion inhibitors operated by the 3-APTES, control samples were also obtained via simple physisorption of MBT and BTA onto unfunctionalized surfaces of MSNPs (MBT-MSNPs and BTA-MSNPs). TGA validated the inclusion of corrosion inhibitors into the MSNP framework. For unfunctionalized MBT-MSNP (Figure S3a) and BTA-MSNP (Figure S3b), the decomposition temperature of MBT and BTA was revealed to be 286 °C with a mass variation of 43% and 245 °C with a mass variation of 29%, respectively. In the fMSNP sample (Figure S3c), a peak at 310 °C with a mass variation of about 12% was attributed to the degradation of 3-APTES. When the corrosion inhibitors are loaded onto fMSNPs, TGA profiles clearly change. For MBT-fMSNP, a weight loss of 4% at 213 °C with a second weight loss of 25% at 356 °C and 417 °C were observed, together with a broad peak at 561 °C (Figure 3a). A less critical change was observed for the TGA profile of BTA-fMSNP (Figure 3b) with two weight losses at 238 °C and 342 °C corresponding to a mass variation of 4% and 8%, respectively. The evident difference in the loading of corrosion inhibitors is probably due to the difference in the chemistry of MBT and BTA [52,53]. MBT is a planar molecule that can be found in two tautomeric forms with a thione (left) or thiol (right) group (Figure 2c). Both tautomeric forms of MBT are reported in the literature to interact strongly with oxidized surfaces. Thione can give a covalent bonding between the exocyclic sulfur atom and the metal site as well as an additional H-bonding between the NH group and surface oxygen atoms [53]. BTA can be present in two different tautomers, the 1H- and the 2H-benzotriazole (Figure 2d). BTA acts as a corrosion inhibitor by forming a metal-BTA surface complex [54]. Such two different ways to prevent the corrosion phenomena could be also responsible for a different loading and release mechanism for the two corrosion inhibitors in the fMSNPs.

The loading of corrosion inhibitors was also validated by measuring changes in the textural properties of engineered MSNPs through N_2_ desorption, reported in Table 3. The presence of 3-APTES drastically induces a decrease in the available surface area of the mesoporous silica nanoparticles from 600 m^2^ g^−1^ to 90 m^2^ g^−1^ with also a decrease in the total pore volume from 1.46 to 0.5 cm^3^ g^−1^. A further substantial decrease was revealed after the loading of the corrosion inhibitor molecules (see Table 3). Regarding the loading efficiency, this was calculated by comparing the theoretical maximum loading capacity with the experimental one for BTA-fMSNP and MBT-fMSNP. Considering that the total pore volume of fMSNP is 0.50 mLg^−1^, and assuming that all the pores are filled with corrosion inhibitor, the maximum theoretical loading capacity was calculated to be 0.68 g(BTA)/1 g (fMSNP) and 0.71 g(MBT)/1 g (fMSNP). On the other hand, the values of loading capacity experimentally obtained from N_2_ desorption for BTA-fMSNP and MBT-fMSNP are 0.23 g (BTA)/1 g (fMSNP) and 0.24 g (MBT)/1 g (fMSNP). BTA (1.36 g mL^−1^) and MBT (1.42 gmL^−1^) density values were used for these calculations. The loading efficiency can be thus estimated to be 34% for both samples, BTA-fMSNP and MBT-fMSNP. The same calculation was used for unfunctionalized MSNPs loaded with corrosion inhibitors to compare the loading efficacy. In Table 3, specific surface area, pore volume, and pore size for the realized samples are presented. As expected, MBT-MSNPs and BTA-MSNPs present a higher loading efficiency than fMSNPs thanks to the lack of 3-APTES molecules.

The entrapment of the corrosion inhibitors in the fMSNPs was also confirmed using SEM-EDS and FTIR. The SEM micrograph in Figure S4a shows a good dispersion of the silica nanoparticles, and the EDS map of sulfur and oxygen (Figure S4b) confirms the homogeneous distribution of MBT in the fMSNP sample.

**Figure 4:**
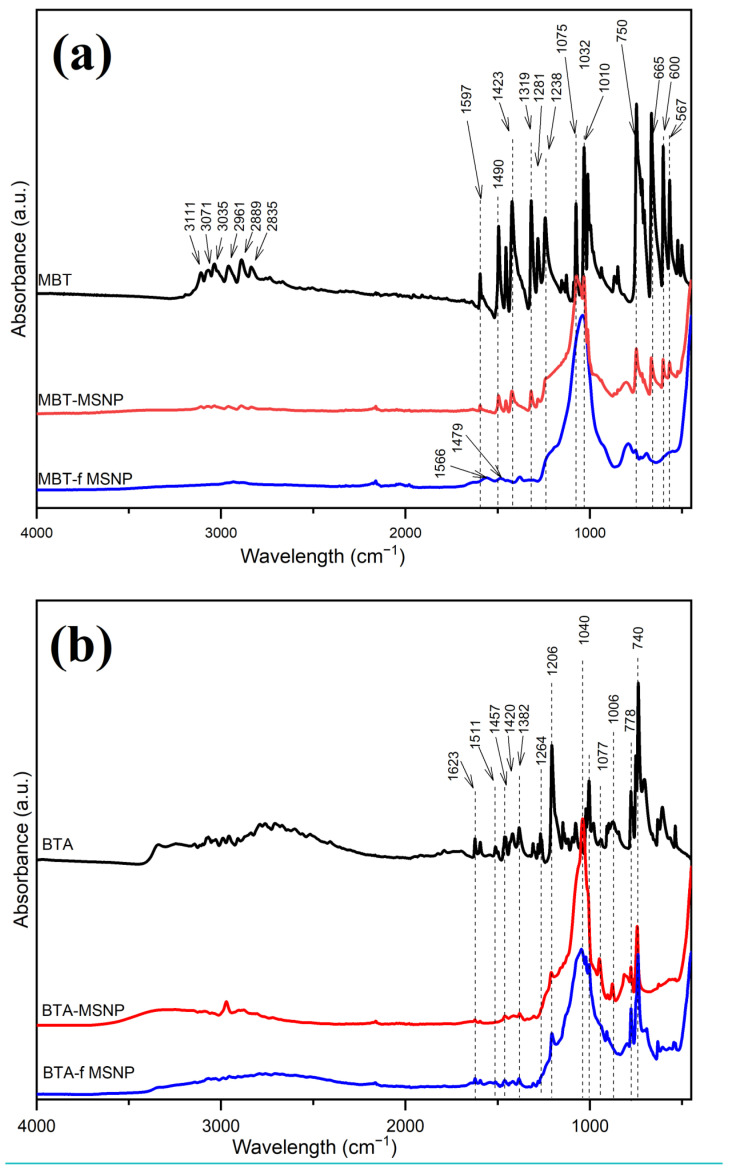
FTIR spectra of (**a**) MBT, MSNPdMBT, and fMSNPdMBT and (**b**) BTA, MSNPdBTA, and fMSNPdBTA.

In Figure 4, FTIR spectra collected in ATR mode of MBT-fMSNP, BTA-fMSNP, MBT-MSNP, and BTA-MSNP are depicted. FTIR spectra of MBT and BTA were taken as a reference. In Figure 4a, the FTIR spectrum of MBT shows peaks in the range 3111–2835 cm^−1^ attributed to the symmetric and asymmetric νNH vibrations. As reported in the literature, νNH can be shifted towards lower wavenumbers due to intermolecular hydrogen bonding when MBT is present as a stable dimer conformer [55]. Peaks observed at 567 and 600 cm^−1^ are associated with aromatic ring torsion. A set of signals in the range 1423–1597 cm^−1^ correspond to νC-C and δNH. The signal at 665 cm^−1^ can be assigned to the νC-S, peaks in the range 1010–1075 cm^−1^ to νC-S in the S-C-S, and bands at 1281 cm^−1^, 1319 cm^−1^, and 1238 cm^−1^ to νC-N. Finally, a strong peak at 750 cm^−1^ is associated with ωCH and ωNH [54]. FTIR spectra of the MBT-MSNP (red line) and MBT-fMSNP (blue line) samples show several peaks attributable to MBT molecules confirming the presence of the corrosion inhibitor on the silica surface. FTIR spectra for MBT-MSNP and MBT-fMSNP are quite similar. MSNPs show characteristic strong and broad peaks at 1100–1049 cm^−1^ and 806 cm^−1^ assigned to the stretching vibrations of the silica framework (νSi–O–Si). Furthermore, the FTIR spectrum of MBT-fMSNP reveals additional peaks at 1566 cm^−1^ and 1479 cm^−1^ assigned to the deformation modes of the amine groups. These groups are highly hydrogen bonded to the silanol groups, forming cyclic structures, as a result of functionalization with 3-APTES [56].

fMSNPs loaded with BTA (Figure 4b) reveal a relevant peak at 1206 cm^−1^ ascribable to the corrosion inhibitor, and it can be attributed to the triazole ring vibration [57]. Some peaks at 1511 cm^−1^ and 1457 cm^−1^ can be assigned to benzene ring deformation, together with additional peaks due to the triazole ring deformation at 1382 cm^−1^ and 1420 cm^−1^. Other characteristic peaks for BTA were reported in Table S2. Also in this case, FTIR spectra of the BTA-MSNP (red line) and BTA-fMSNP (blue line) samples show a large number of peaks that confirm the presence of BTA on the silica surface. The spectrum of the BTA-fMSNP sample shows characteristic peaks of 3-APTES at 1566 cm^−1^ and 1479 cm^−1^ assigned to the NH_2_ deformation. In Table S2, FTIR characteristic signals for all the samples were summarized.

### 3.3. Corrosion Inhibitor Release

**Figure 5:**
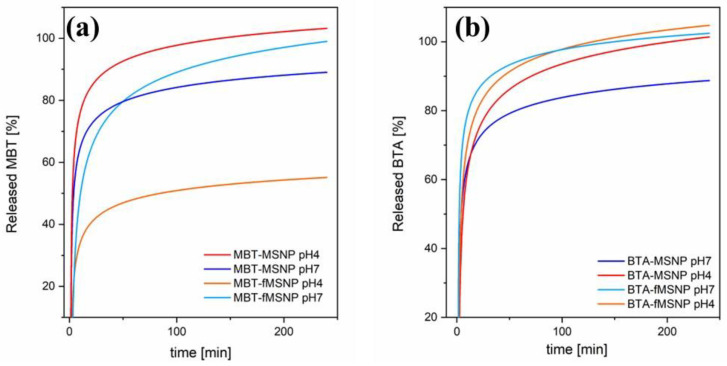
(**a**) MBT and (**b**) BTA release curves from MBT-MSNP, BTA-MSNP, BTA-fMSNP, and BTA-fMSNP at pH 7 and pH 4 for the first 250 min.

Typically, the presence of acid conditions on metal surfaces can be a potential trigger for corrosion processes. However, in some cases (e.g., copper alloys in the presence of chloride ions) degradation may also occur under neutral pH conditions [2]. The effects of the engineering of the mesoporous silica network on the release properties were investigated by comparing the MBT and BTA release kinetics from MBT-fMSNP, BTA-fMSNP, MBT-MSNP, and BTA-MSNP samples (Figure 5). The release of corrosion inhibitors was studied at pH 7 and pH 4 for the first 4 h, through UV-Vis spectroscopy. The amount of corrosion inhibitor released in the solution was monitored for 240 min at regular intervals. A logarithmic fit was used to represent the kinetic curves, and the values used for fitting were normalized to the mass of corrosion inhibitors determined with TGA.

Figure 5a shows the release kinetic curves for MBT-MSNP and MBT-fMSNP at pH 4 and pH 7. The profiles obtained at different pH values clearly show different behaviors revealing characteristic kinetic trends. In general, MBT-MSNP samples show an initial fast release (80–90%) reaching the plateau after the first hours at pH 4 and pH 7. It is evident that when the MSNPs are in an acid solution, almost 100% of MBT is released within the first 4 h. On the contrary, MBT-fMSNP samples reveal a two-stage mechanism of release. An initial fast release is followed by a second stage, characterized by slow kinetics. It is interesting to note that in acidic conditions, a fraction of MBT (40–50%) is released within the first hour. It is probably due to the presence of physisorbed MBT on the silica surface. After 60 min, the rest of the MBT is gradually released with significantly slower kinetics compared to the unfunctionalized silica nanoparticles; considering the slope of the curve, the complete release of the MBT molecules could be accomplished in a much longer lapse of time. Further studies on the release mechanism of a longer timescale will be published elsewhere. At pH 7, a higher amount of MBT (80–90%) is released in the solution but, also in this case, with slower kinetics compared to MBT-MSNP. The gradual release of the corrosion inhibitor operated by the MBT-fMSNP samples can be explained with a pH-driven open/close mechanism that arises from the hydrogen bonding interaction between amines at neutral pH (open gate) and coulombic repulsions at acidic pH between closely located 3-APTES molecules at the pore openings (closed gate). When MBT-fMSNPs are protonated in acidic conditions, the amine chains tend to be rigid and push themselves away from the pore openings, inhibiting the release of MBT.

Figure 5b shows the release profile of BTA-MSNP and BTA-fMSNP at pH 4 and pH 7. The profiles obtained at different pH values are quite similar in behavior for BTA-MSNP and BTA-fMSNP. In both neutral and acidic conditions, and for both functionalized and unfunctionalized MSNPs, a large part of BTA is released during the first 4 h. It is evident that the mechanism of pH-dependent release is less efficient for the system BTA-fMSNP.

As a general consideration, the synthesized MSNPs grant a gradual release of the corrosion inhibitors on a timescale of several hours. This can be explained either by the high loading efficiency of the MSNPs induced by the peculiar bicontinuous concentric lamellar morphology, both for the fact that MSNPs were functionalized with 3-APTES. Similar systems are reported in the literature where silica nanoparticles with different shapes and structures were engineered for a gradual or controlled release of MBT and BTA. As can be seen in Table 4, silica nanocontainers are characterized by a high percentage of loading efficiency (50–99%) and slow release (from some hours to some days), but the outflow of the active molecules is usually not triggered. On the other hand, in the functionalized silica particles where the release of the guest molecules is triggered by physical or chemical inputs, a maximum release time ranging from a few hours to a couple of days was obtained. In this work, MBT was successfully loaded in MSNPs and fMSNPs with good efficiency values of 65% and 35%, respectively. Furthermore, a prolonged release time of several hours, was achieved for MBT-fMSNPs in an acid solution, mimicking a corrosive environment. Regarding BTA, this was efficiently loaded in the mesoporous framework of silica, but its release seems to not be triggered by the change in pH. However, for both systems BTA-MSNP and BTA-fMSNP, the guest molecules are dispersed in the aqueous solution in a time frame of 4–5 h.

## 4. Conclusions

Mesoporous dendritic fibrous silica nanoparticles with a very large surface area and pore volume were synthesized with a high yield (86%). Taking advantage of their peculiar morphology, silica nanoparticles were successfully functionalized with 3-APTES. This organosilane coupling agent can work with a cage-like mechanism, entrapping and releasing functional molecules in a controlled manner. Engineered silica nanoparticles were then loaded with MBT and BTA as corrosion inhibitors. Release studies of MBT revealed that the functionalization of the nanocarrier helps to retain the anti-corrosive agent for a longer period (several hours) compared to the simple physisorption of the active molecule onto the mesoporous silica surface (a couple of hours). A prolonged release time was achieved for the MSNPs functionalized with 3-APTES and loaded with MBT when immersed in an acid solution. The acidic medium was used to simulate the corrosive environment. On the contrary, the release of BTA was not triggered by the change in pH; however, thanks to the peculiar dendritic structure of the synthesized silica nanoparticles, the guest molecules were slowly diffused in the aqueous medium for 4–5 h. Such promising results pave the way for the use of functionalized mesoporous silica nanoparticles in polymeric films for the long-term protection of metals against corrosion phenomena.

## Figures and Tables

**Table 1:** Description of the synthesized samples.

Name	Description
MSNP	Mesoporous Silica NanoParticle
MBT-MSNP	MSNP + physisorbed MBT
BTA-MSNP	MSNP + physisorbed BTA
fMSNP	3-APTES-functionalized MSNP
MBT-fMSNP	fMSNP + entrapped MBT
BTA-fMSNP	fMSNP + entrapped BTA

**Table 2:** TEM and SEM images, synthesis protocol, yield, and average particle size of the synthetized MSNPs.

Sample	TEM Image	SEM Image	Synthesis	Yield [%]	Average Size Particles [nm]
K-3	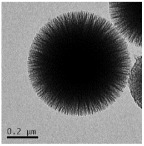	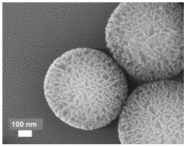	W/O ratio 1:1 hydrothermal method 4 h	41	700
K-6	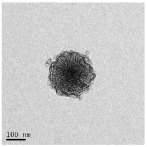	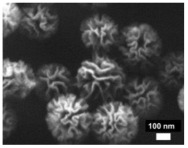	W/O ratio 1:1 hydrothermal method 2 h	≈4	325
K-7	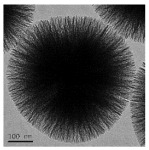	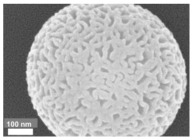	W/O ratio 1:2.3 hydrothermal method 4 h	≈60	630
K-9	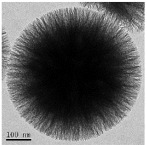	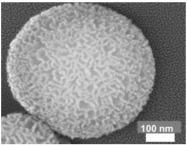	W/O ratio 1:2.3 hydrothermal method 16 h	≈80	630
K-4	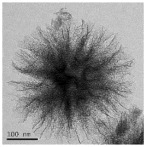	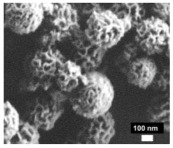	W/O ratio 1:1 reflux method 24 h	40	350
K-5	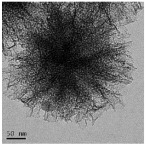	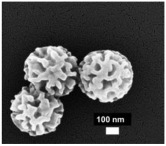	W/O ratio 1:1 reflux method 48 h	86	450

**Table 3:** Specific surface area, pore volume, and pore size from N_2_ physisorption for all samples.

Sample	BET S.A.	Pore Volume	Pore Size	Loading Efficiency
	[m^2^ g^−1^]	[cm^2^ g^−1^]	[nm]	[%]
MSNP	592	1.46	8.74	-
BTA-MSNP	270	0.71	9.63	51
MBT-MSNP	133	0.53	14.96	64
fMSNP	90	0.50	22.77	-
BTA-fMSNP	50	0.33	25.95	34
MBT-fMSNP	41	0.33	32.62	34

**Table 4:** Characteristic properties of silica nanocarriers for MBT and BTA delivery.

Ref.	Silica	Corrosion Inhibitor	Max Time Release [min]	Trigger	Functionalities between MSN and Corrosion Inhibitor	Loading Efficiency
[58]	hollow	BTA	30	no	-	52–99%
[59]	hollow	MBT	1500	no	-	20%
[47]	functionalized	BTA	200	pH	CuSO_4_	43%
[60]	hollow	MBT	600	no	-	72%
[16]	hollow	MBT	12,000	no	-	68%
[43]	functionalized	BTA	3600	pH	AEPTS	12%
[61]	functionalized	BTA	420	UV light	ICPES	25%
[27]	functionalized	MBOH	3000	pH	AP	25%
[28]	functionalized	BTA	100	pH	CuSO_4_	≈13%
[29]	functionalized	BTA	-	-	OTES	-
[62]	functionalized	MBT	1500	pH	piperazine	13%
[30]	functionalized	BTA	120	pH	CPTES, BDA, CB	64%

AEPTS: 3-[2-(2-aminoethylamino)ethylamino]propyl-trimethoxysilane; ICPES: isocyanatopropyltriethoxysilane; AP: γ-aminopropyltriethoxysilane; MBOH: 2-Mercaptobenzoxazole; OTES: octyltriethoxysilane; CPTES: 3-chloropropyltriethoxysilane; BDA: 1,4-butanediamine; CB: Cucurbit.

## Data Availability

Data are contained within the article or Supplementary Materials.

## Supplementary Materials

The following supporting information can be downloaded at https://www.mdpi.com/article/10.3390/nano13182543/s1, Table S1: table with the release time before the drawing of the supernatant for the MSNP-based samples; Table S2: functional groups and mode of vibration from ATR-FTIR spectra of all samples; Figure S1: UV-Vis calibration curve of BTA (a) and MBT (b); Figure S2: DLS measurement of MSNP and fMSNP; Figure S3: TGA (dash line) and DTG (solid line) of (a) MBT-MSNP, (b) BTA-MSNP, and (c) fMSNP; Figure S4: SEM-EDS analysis of MBT-fMSNPs: (a) SEM image and (b) EDS oxygen—sulphur overlapped maps.
