# Supplementary material for: 3-APTES on Dendritic Fibrous Mesoporous Silica Nanoparticles for the pH-Controlled Release of Corrosion Inhibitors
Source: Nanomaterials (Basel). 2023 Sep 11;13(18):2543. doi: 10.3390/nano13182543 (PMC10537782; doi:10.3390/nano13182543)
Supplement: Supplementary file 1 [file nanomaterials-13-02543-s001.zip › nanomaterials-2579489-supplementary.pdf]

3-APTES on Dendritic Fibrous Mesoporous Silica Nanoparticles for the pH Controlled Release of Corrosion Inhibitors

E. Marconi [1,2,3], I. Luisetto [4], G. Di Carlo [5], M. P. Staccioli [5], S. Tuti [3], and L. Tortora [1,2,3*]

1 LASR3 Surface Analysis Laboratory Roma Tre, via della Vasca Navale 84, Rome, 00146, Italy
2 National Institute for Nuclear Physics, INFN Roma, Tre via della Vasca Navale 84, Rome, 00146, Italy; eleonora.marconi@roma3.infn.it
3 Department of Sciences, Roma Tre University, via della Vasca Navale 84, Rome, 00146, Italy; simonetta.tuti@uniroma3.it, luca.tortora@uniroma3.it
4 Department of Energy Technologies, ENEA C.R. Casaccia DTE-PCU-IPSE, S.P. 081 Via Anguillarese 301, 00123 S.M. di Galeria, Rome, Italy; igor.luisetto@enea.it
5 Institute for the Study of Nanostructured Materials, National Research Council (ISMN-CNR), Via Salaria km 29,3 Monterotondo, Rome, 00015, Italy; mariapaola.staccioli@ismn.cnr.it, gabriella.dicarlo@cnr.it
* Correspondence: luca.tortora@uniroma3.it

Table S 1: Table with the release time before the drawing of the supernatant for the MSNP-based samples.

**SAMPLES: BTA-MSNP, BTA-fMSNP, MBT-MSNP, MBT-fMSNP**								
**RELEASE TIME**	T_0	T_0+5min	T_0+30min	T_0+60min	T_0+120min	T_0+180min	T_0+240min	T_0+24h

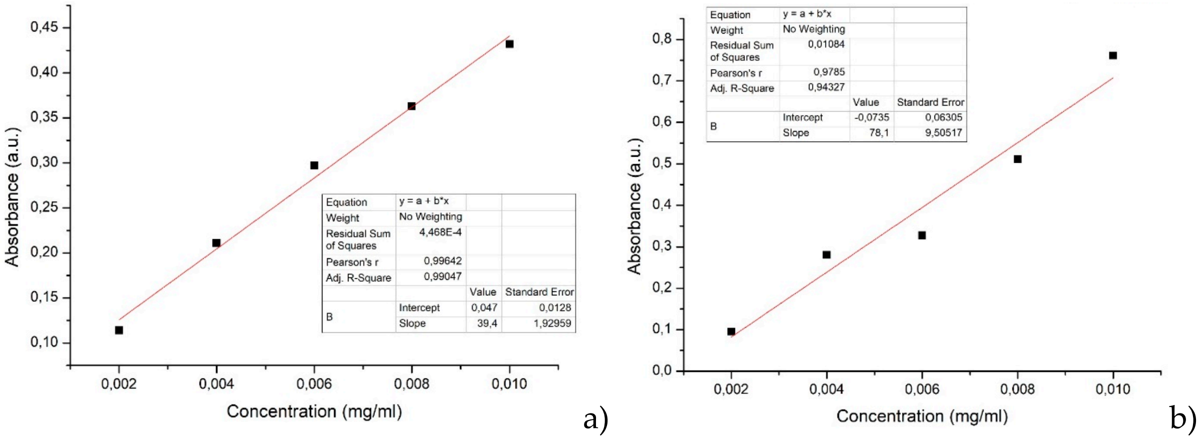

Figure S1: UV-Vis calibration curve of BTA a) and MBT b).

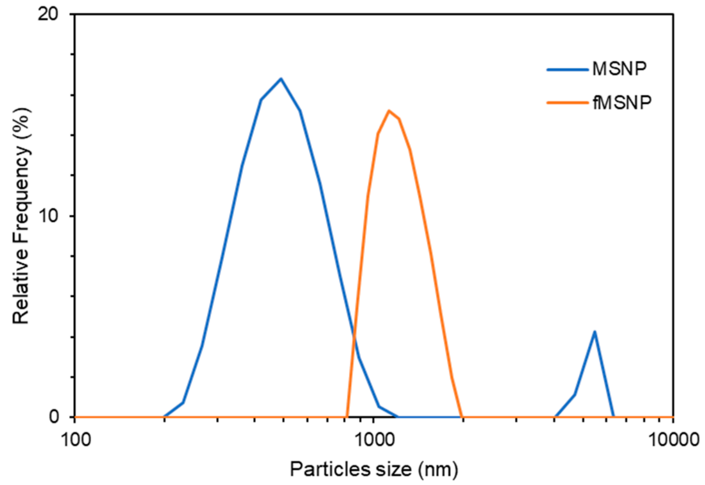

Figure S2: DLS measurement of MSNP and fMSNP.

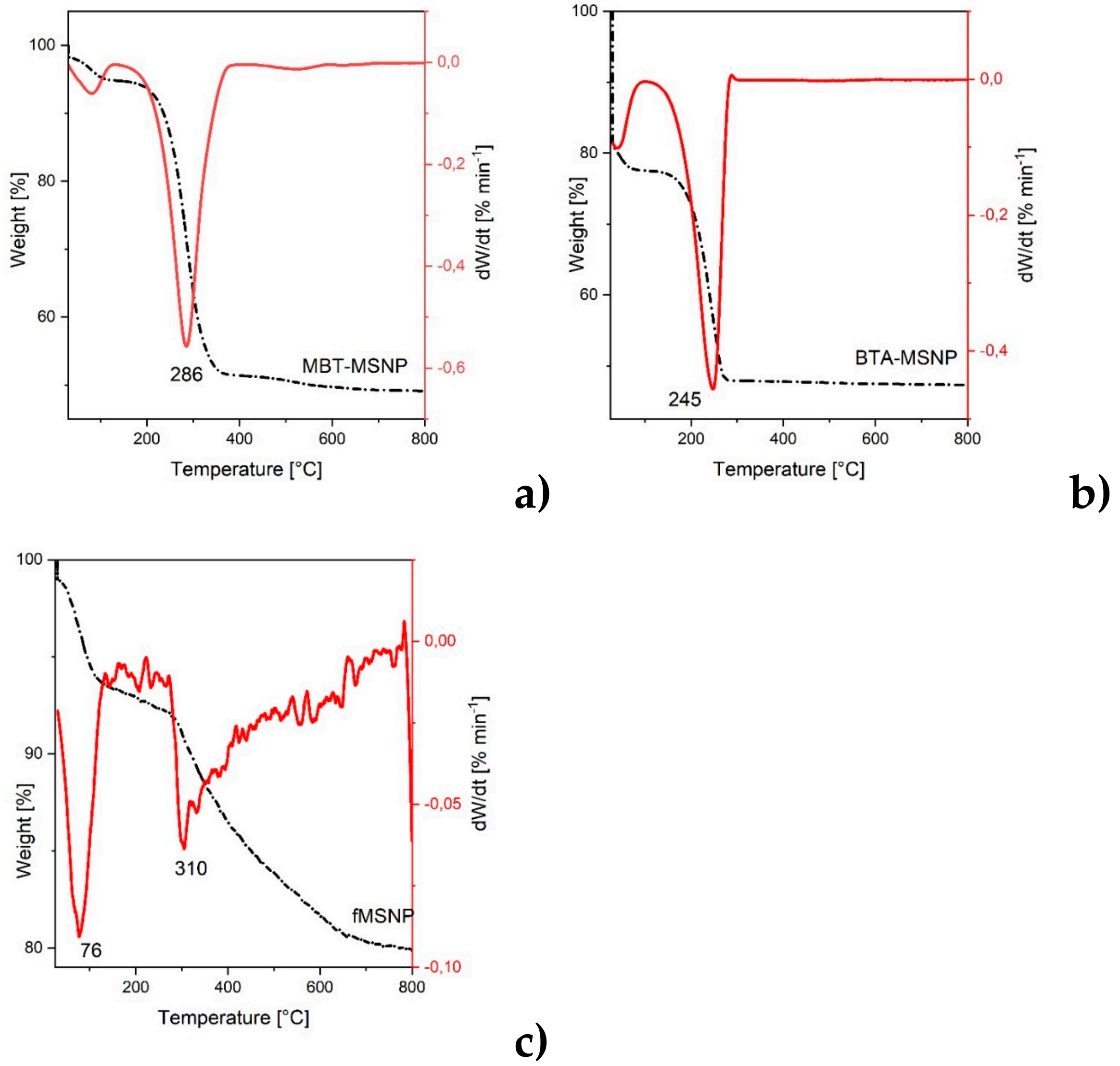

Figure S3: TGA (dash line) and DTG (solid line) of a) MBT-MSNP b) BTA-MSNP, and c) fMSNP.

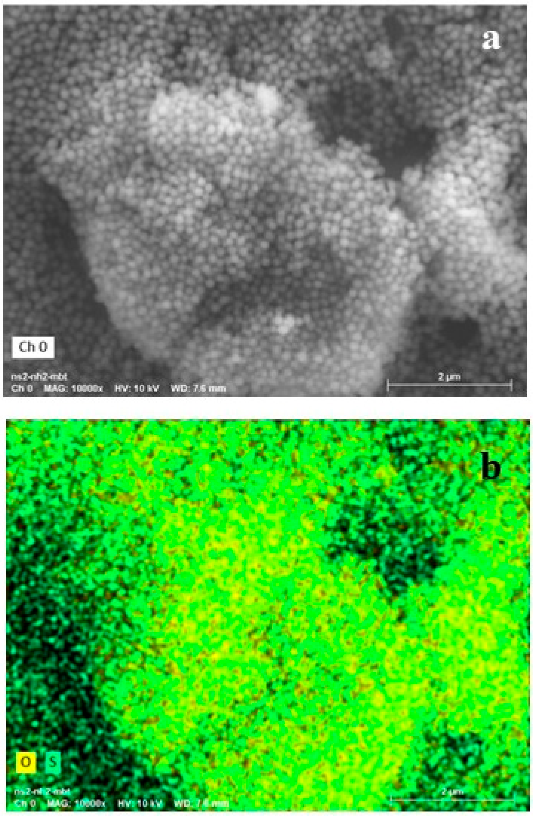

Figure S4: SEM-EDS analysis of MBT-fMSNPs: a) SEM image b) EDS oxygen - sulphur overlapped maps.

Table S2: Functional groups and mode of vibration from ATR-FTIR spectra of all samples

Sample	Wave number (cm^{-1})	Vibration type	Assignment
MSNP	1100-1049	Stretching	n_sSi-O-Si
	806	Stretching	n_{as}Si-O-Si
fMSNP	1479; 1566	Bending	dNH_2
	2871	Stretching	n_sCH_2
	2934	Stretching	$n_{as}CH_2$
MBT	567; 600	Aromatic ring torsion	Aromatics
	665	Stretching	nC-S
	750	Wagging	wCH and wNH
	1010 - 1075	Stretching	nC-S in the S-C-S
	1281; 1319; 1238	Stretching	nC-N
	1423	Stretching	nC-C
	1597	Bending	dNH
	3111 – 2835	Stretching	nN-H
MBT- MSNP	567; 600	Aromatic ring torsion	Aromatics
	665	Stretching	nC-S
	750	Wagging	wC-H
	1319 - 1238	Stretching	nC-N
	1423	Stretching	nC-C
	1597	Bending	dN-H
	3100-2800	Stretching	nN-H
MBT- fMSNP	750; 725	Stretching	nC-S
	1479; 1566	Bending	dNH_2
BTA	778	In plane bending	d N-H
	1006; 740	out-of-plane bending	g C-H
	1206	triazole ring vibration	aromatics
	1264	Stretching	nC-N
	1382; 1420	triazole ring deformation	aromatics
	1457; 1511	benzene ring deformation	aromatics
	1623; 1077	Bending	dNH
	3078-3032	Stretching	nCH
	3400-3200	Stretching	nNH
BTA- MSNP	778-740	out-of-plane bending	g C-H
	1206	triazole ring vibration	aromatics
	1382	triazole ring deformation	aromatics
BTA- fMSNP	778	In plane bending	d N-H
	740	out-of-plane bending	g C-H
	1206	triazole ring vibration	aromatics
	1382-1457	Bending	dNH_2
	1479; 1566	Bending	dNH_2
